# Supplementary material for: Characterization of Cme and Yme thermostable Cas12a orthologs
Source: Commun Biol. 2022 Apr 6;5:325. doi: 10.1038/s42003-022-03275-2 (PMC8986864; doi:10.1038/s42003-022-03275-2)
Supplement: Supplementary file 2 — Supplementary Information [file 42003_2022_3275_MOESM2_ESM.pdf]

SUPPLEMENTARY FIGURES

Figure S1. Formation of Cas12a ribonucleoprotein complexes.

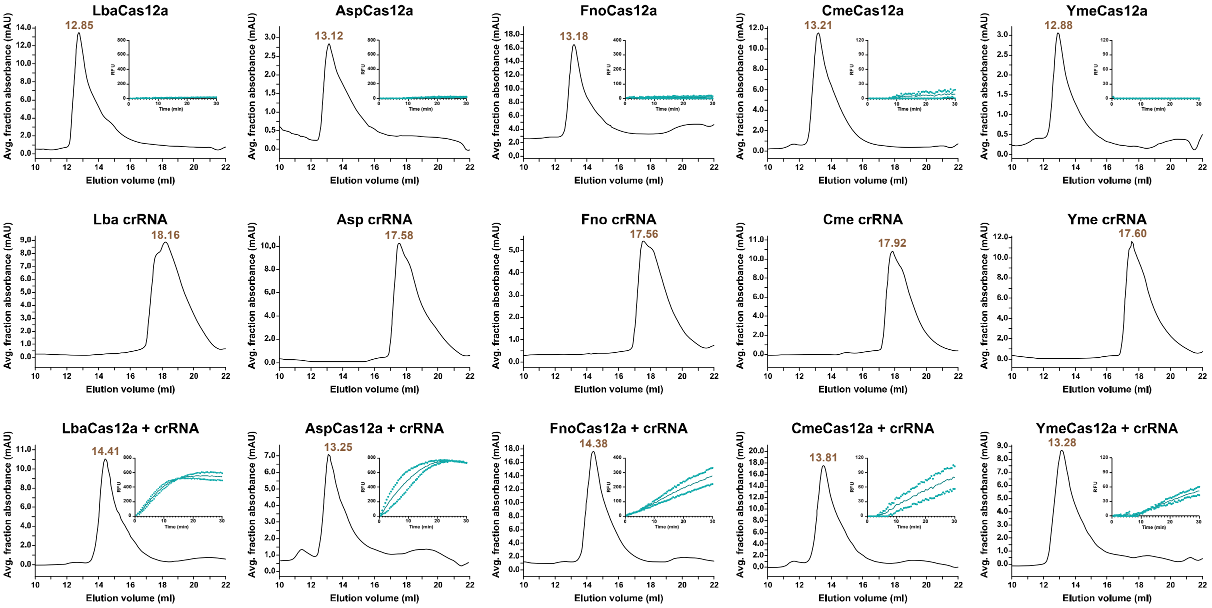

Size exclusion chromatography was used to monitor the formation of Cas12a RNP complexes. Samples consisting of Cas12a protein without crRNA, crRNA without Cas12a protein or an equimolar mixture of Cas12a protein with crRNA were separated on a size exclusion column. Representative chromatographs show absorbance at 280 nm during the course of elution. Elution volume at the peak of absorbance is labelled in brown above the peak. Shown inset for Cas12a protein without crRNA or Cas12a protein with crRNA is the target DNA-activated *trans* nuclease activity of the concentrated fractions corresponding the elution peak as monitored by cleavage of a fluorescent reporter oligonucleotide over time. A line is plotted showing the mean relative fluorescent unit (RFU) values for reactions. The RFU values represent background normalized signal derived by subtracting the fluorescence of negative control reactions that did not contain target DNA. Individual data points for two experimental replicates are plotted on the graphs and the source data can be found in the supplementary materials.

Figure S2. Raw traces of nanoDSF assays.

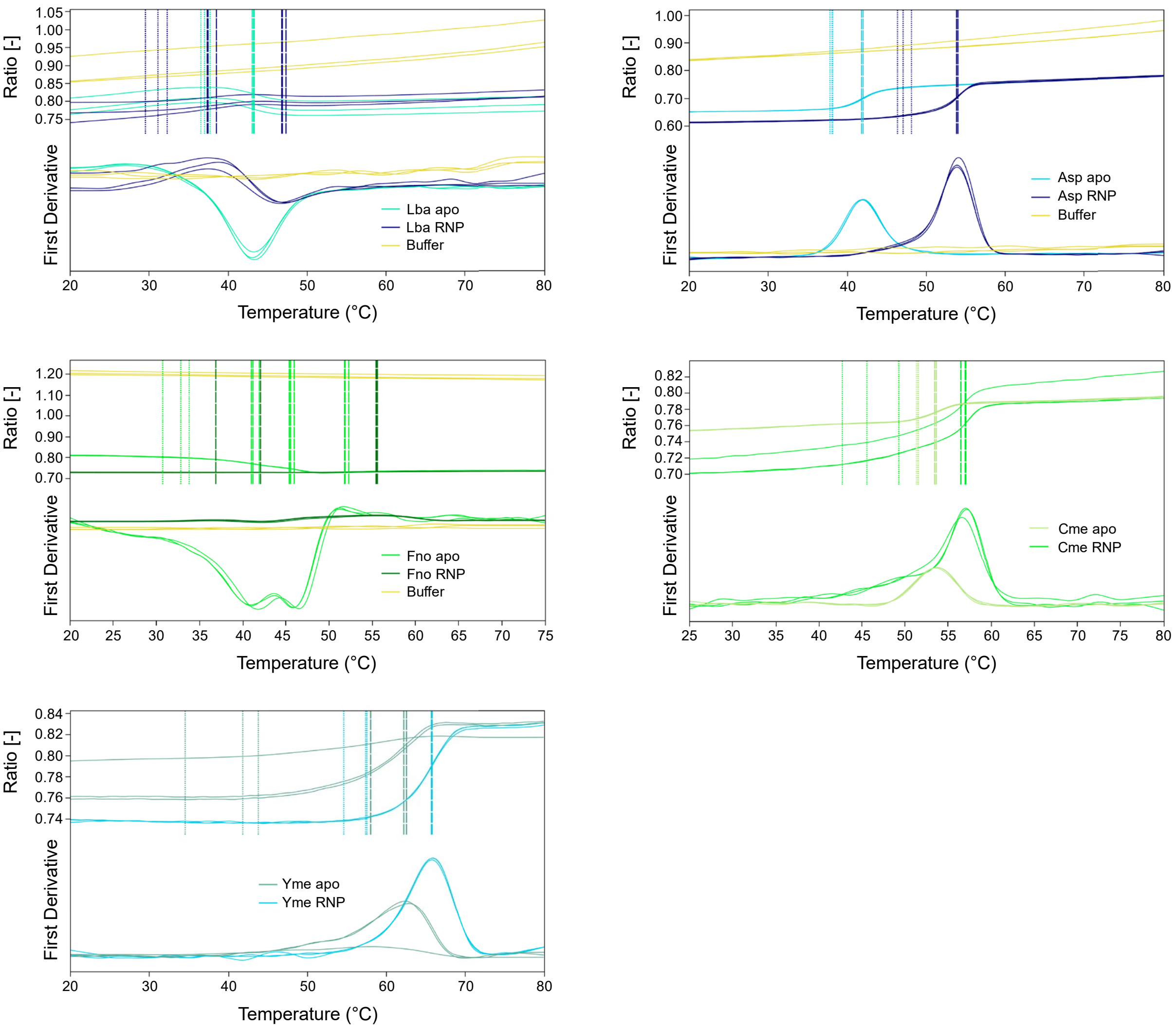

The fluorescence ratio and first derivative were graphed by the Prometheus instrument during experiments where the temperature was increased at the rate of 1 °C per second for Cas12a protein without crRNA (apo), with crRNA (RNP) or reaction buffer (buffer). The peaks in the first derivative graph are inflection points that correspond to the temperature at which half of the molecules have undergone transition to a new state, which is interpreted as the protein unfolding (denaturing or melting).

Figure S3. Workflow for PAM preference assays.

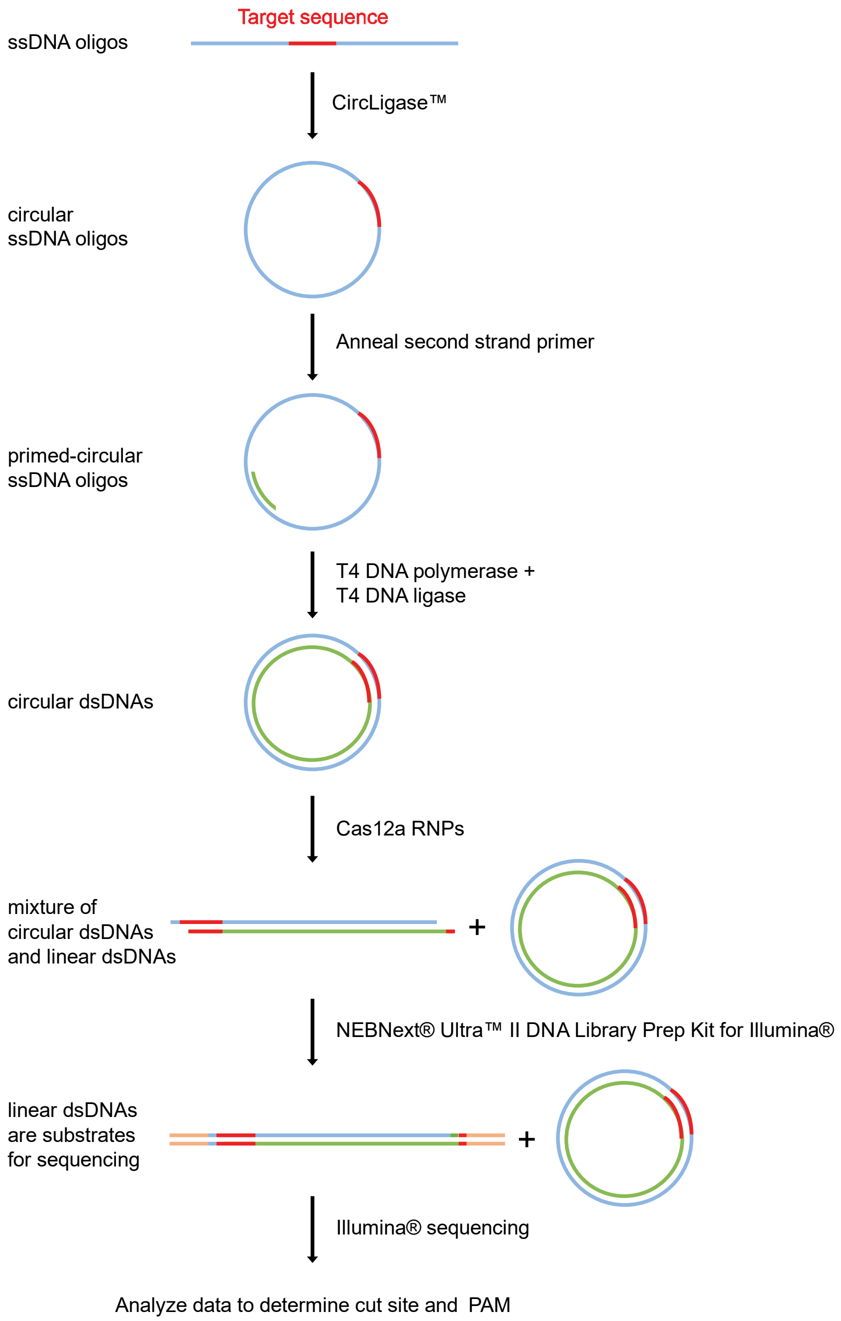

A pool of single-stranded DNA oligos containing a 10 nt randomized region (NNNNNNNNN; red) adjacent to a target sequence were converted into circular double-stranded DNA by the method diagrammed above. After exposure to crRNA-Cas12a RNPs, linearized DNA is a substrate for adapter ligation and high-throughput sequencing while uncut circular DNA is not.

Figure S4. PAM preference of Cas12a orthologs using Target D.

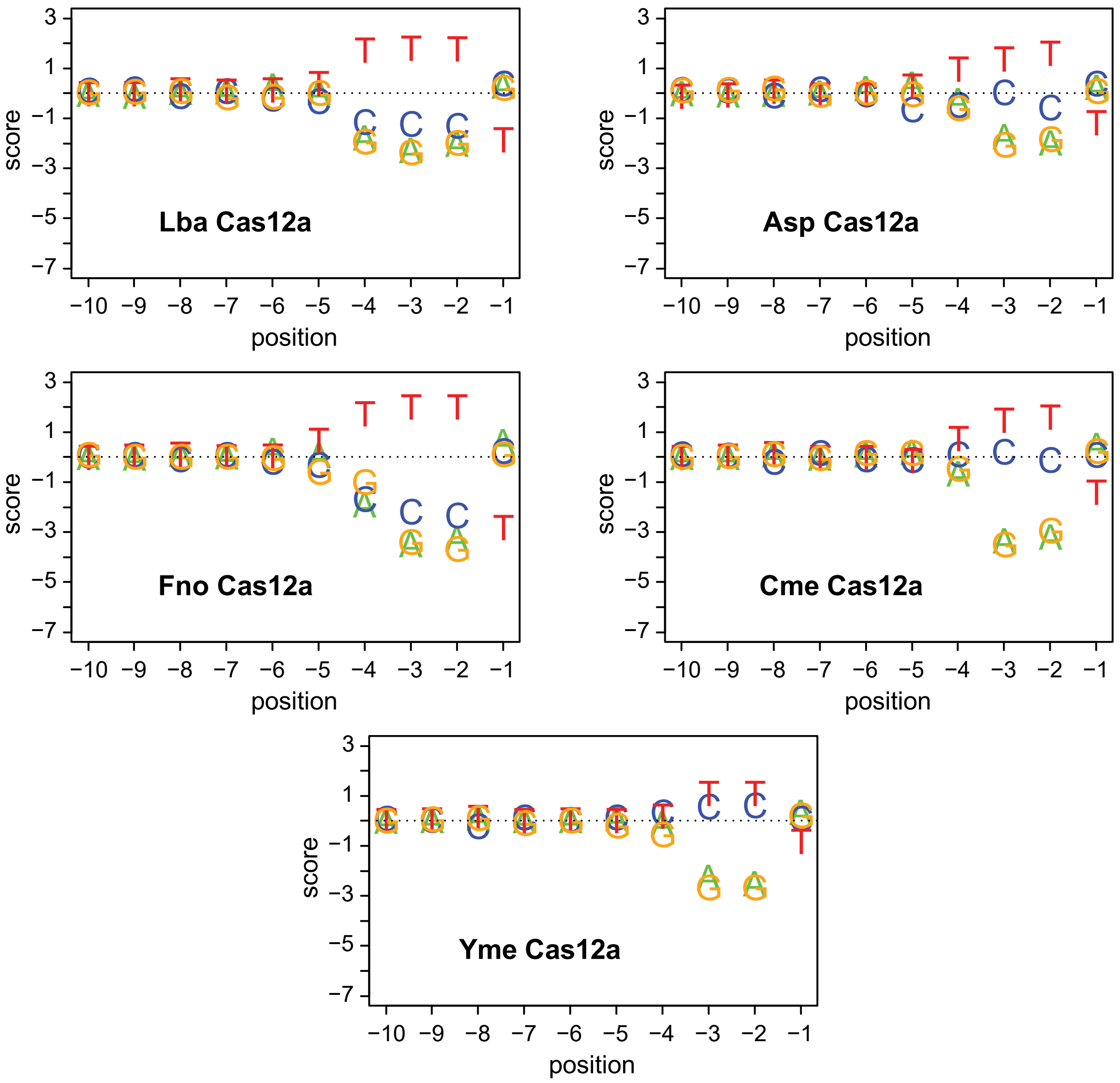

A step-wise diagram of the method used to generate this data is provided in Supplementary Figure S3. Circular, double-stranded DNA libraries were linearized by Cas12a RNPs and subjected to end repair, adapter ligation and high-throughput sequencing. Position weight values were calculated as described in the methods and are shown as graphs where a positive score corresponds to enrichment of a particular base in the randomized 10 nt region on a log_2 scale and a negative score corresponds to depletion. Position is the distance in nt relative to the first nt of target sequence. Sequences of oligonucleotides used to make circular substrate DNAs can be found in Supplementary Table S1.

Figure S5. Tolerance of non-canonical PAMs by Cas12a orthologs.

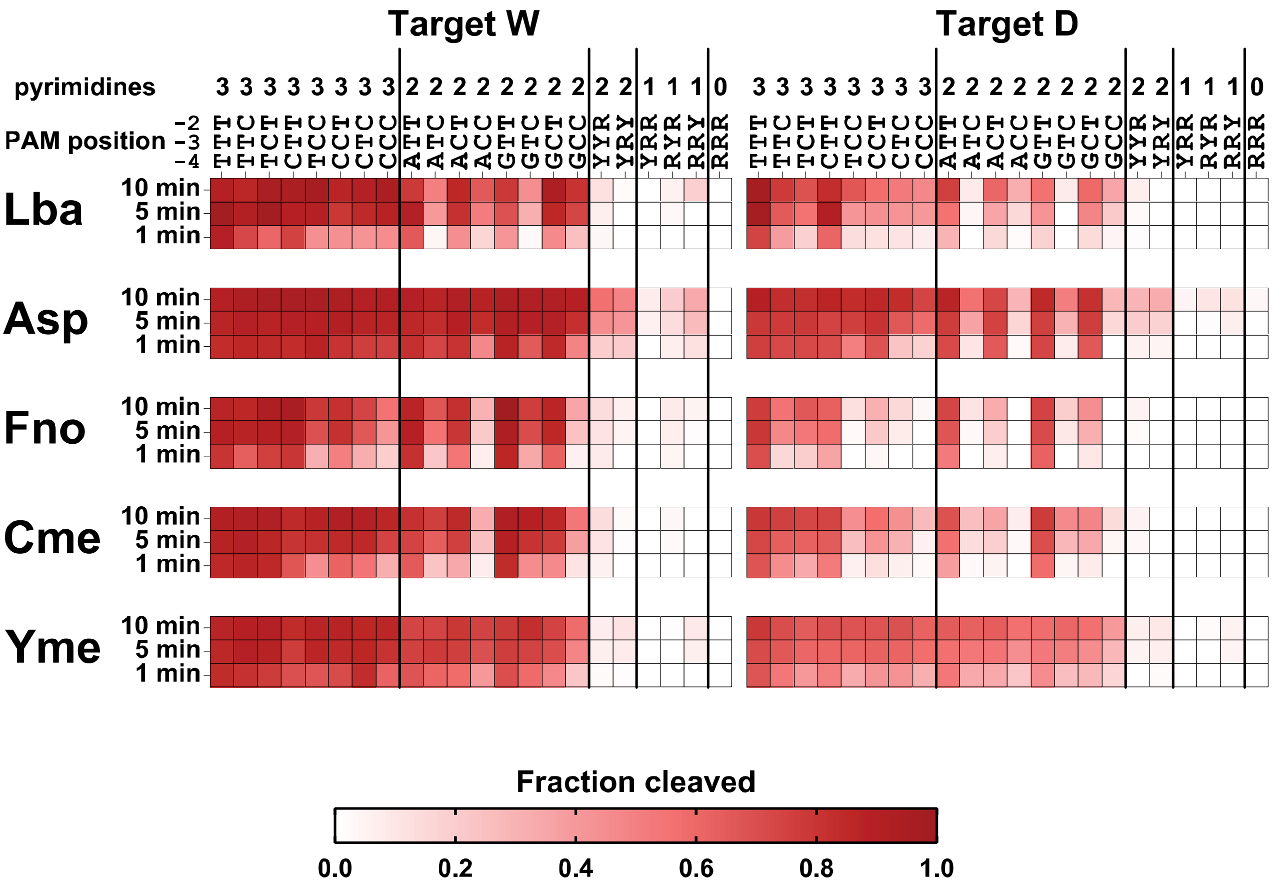

Target-directed double-stranded DNA (dsDNA) cleavage activities of Cas12 RNPs were measured using *in vitro* assays containing fluorophore-labelled dsDNA target substrates with variant PAM sequences. Target DNAs were identical except for nucleotide identity at the -2, -3 and -4 positions relative to the first nucleotide of the target sequence. Aliquots from cleavage reactions were removed and quenched after 1, 5, 10 min. Cleaved fragments were quantified and the proportion of the substrate cleaved in each condition is represented in a heat map where the intensity of the red color corresponds to the extent of target cleavage. Reactions were performed at 37 °C for Lba, Asp, and Fno while Cme and Yme reactions were performed at 55 °C. The source data used to make the graphs can be found in the supplementary materials.

Figure S6. Results of cleavage site assays using restriction enzymes as controls.

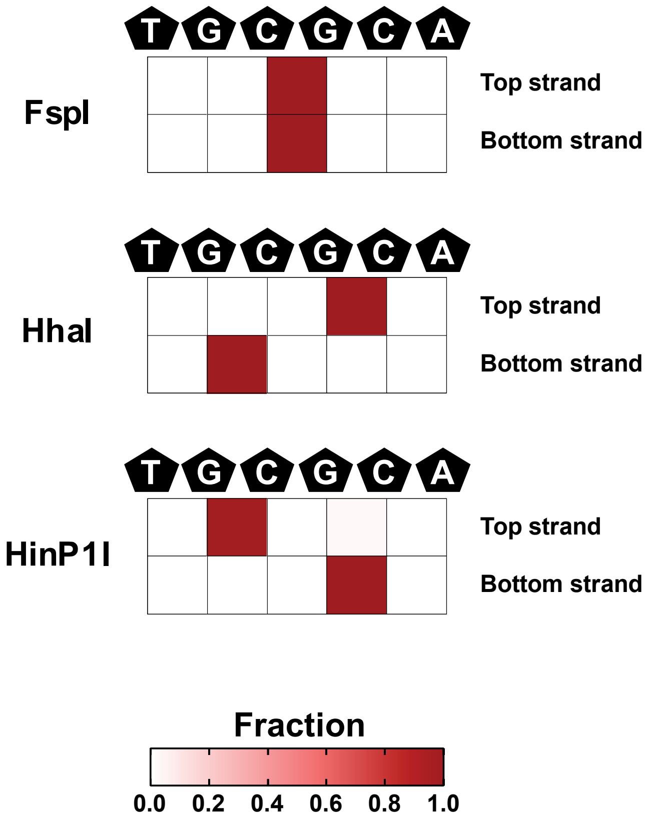

Circular double-stranded DNA containing a TGCGCA recognition sequence for FspI, HhaI, and HinPI1 restriction enzymes was made using the method described in Supplementary Figure S3. Reactions were set up where the circular DNA was incubated with one of the three restriction enzymes and subjected to end repair, adapter ligation and high-throughput sequencing. The resulting DNA was sequenced end to end in both reads of a paired-end sequencing run and custom scripts were used to map the cleavage site on the top strand and bottom strand. The diagrams in this figure show the summation of cleavage position frequencies. All three enzymes produced their expected cleavage profile; blunt for FspI, 2 nt 3' overhang for HhaI, and 2 nt 5' overhang for HinP1I. Full data sets with matrices of the frequency of each top strand and bottom strand cleavage site combination are available in supplementary materials. The sequences of the oligonucleotides used to make circular substrate DNA can be found in Supplementary Table S1.

Figure S7. Profile of cleavage sites on target DNA generated by Cme and YmeCas12a at 37 °C and 55 °C.

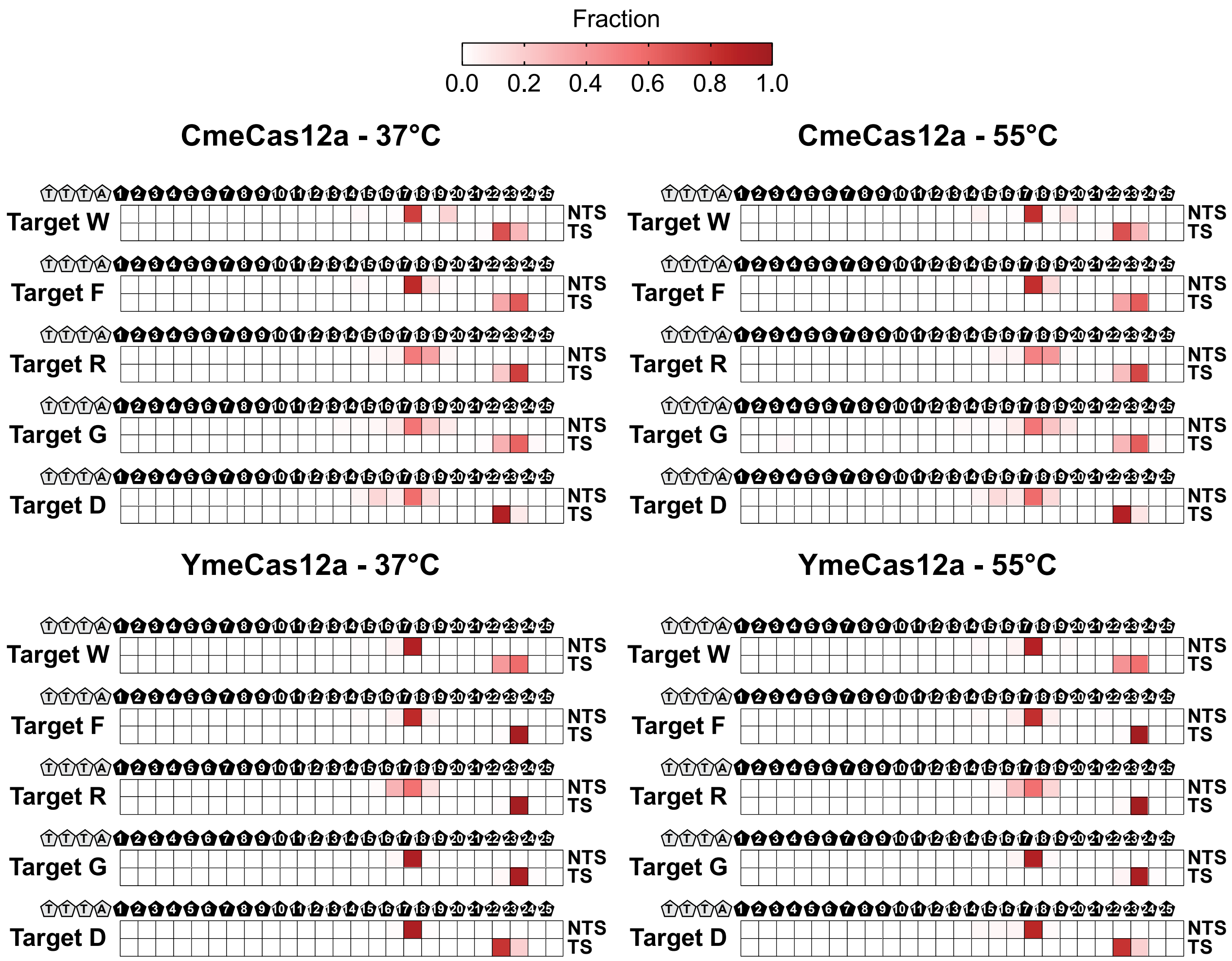

Circular double-stranded DNA containing a TTTA PAM sequence flanked by a target sequence was made using the method described in Supplementary Figure S3, incubated with a complementary crRNA-Cas12a RNP and subjected to end repair, adapter ligation and high-throughput sequencing. The substrate DNA was sequenced end to end in both reads of a paired-end sequencing run and custom scripts were used to map the cleavage site on the non-target strand (NTS) and target strand (TS). The diagrams in this figure show the individual summation of all NTS cleavage position frequencies and TS cleavage position frequencies. Full data sets with matrices of the frequency of each NTS and TS cleavage site combination are available in the supplementary materials. Sequences of crRNAs and oligonucleotides used to make circular substrate DNAs can be found in Supplementary Table S1.

Figure S8. Controls for *trans* nuclease assays.

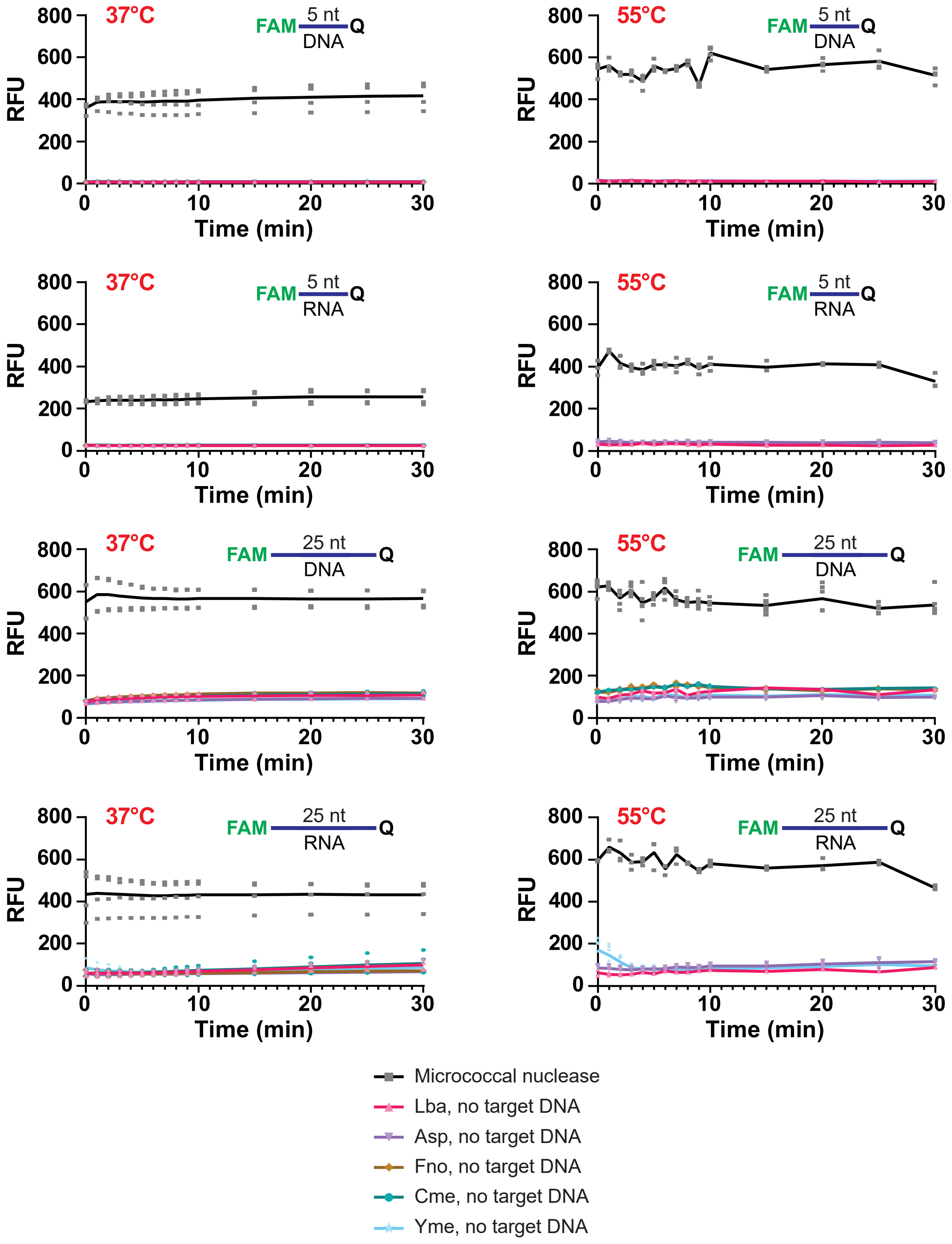

5' fluorophore and 3' quencher labelled DNA or RNA oligonucleotides with either 25 nt of defined sequence or 5 nt of randomized sequence were incubated in reaction buffer with either micrococcal nuclease or a Cas12a RNP without target DNA present at 37 °C or 55 °C. Fluorescence was

measured over time and mean relative fluorescent unit (RFU) values are plotted as lines. Individual data points for all experimental replicates are plotted on the graphs and the source data can be found in the supplementary materials.

Figure S9. Gel electrophoresis of labelled ssDNA after exposure to Cas12a RNPs.

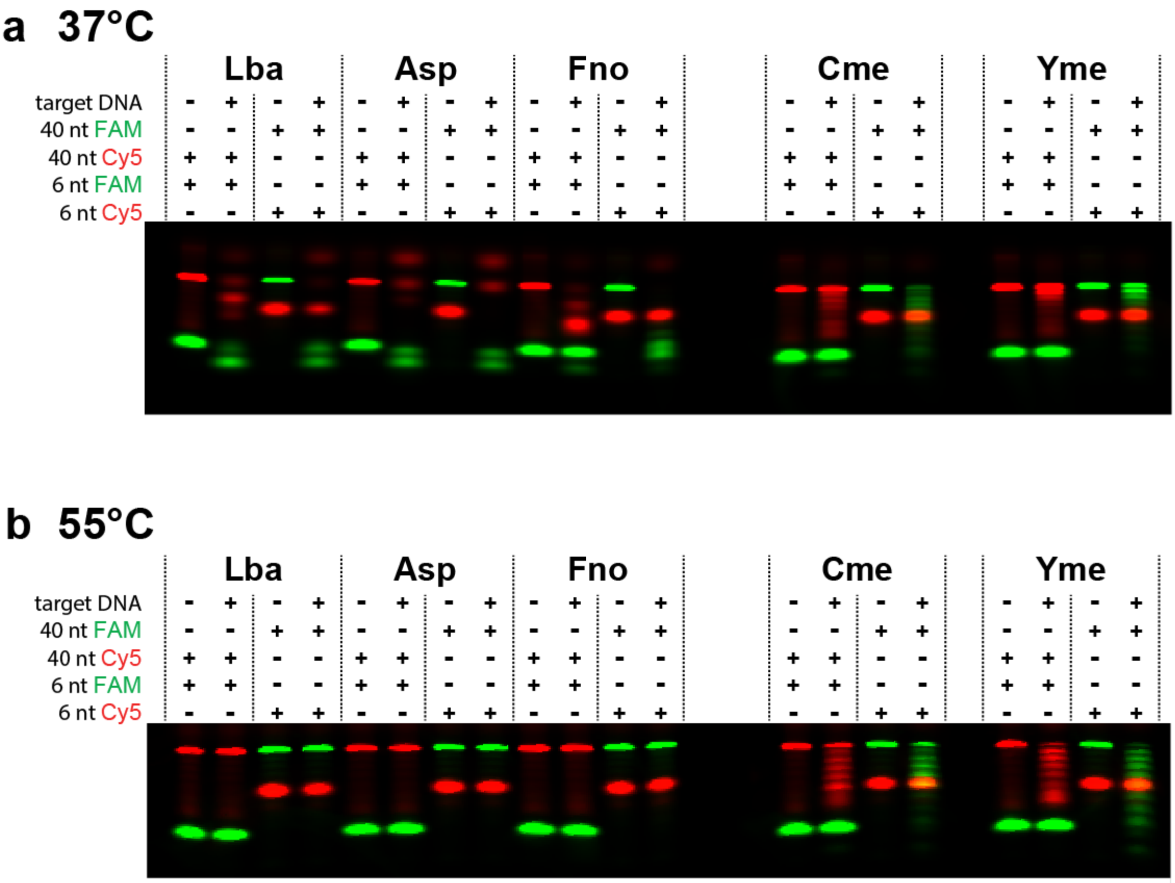

Cas12a was incubated with crRNA at 25 °C for 10 min to form RNPs. The RNPs were incubated at 37 °C **(a)** or 55 °C **(b)** for 5 min prior to addition of target DNA that had also been incubating at that temperature. After 10 min, a mixture of 5' fluorescein (FAM) and 5' Cy5 single-stranded DNAs either 6 nt or 40 nt in length was added and incubation continued for an additional 30 min. Reactions were quenched with EDTA and Proteinase K and separated on a 15% TBE-Urea gel. Labelled DNAs were visualized using a Typhoon™ Variable Mode Imager (GE Healthcare, NJ, USA).

Figure S10. Comparing poly T to balanced sequence *trans* nuclease activity reporters.

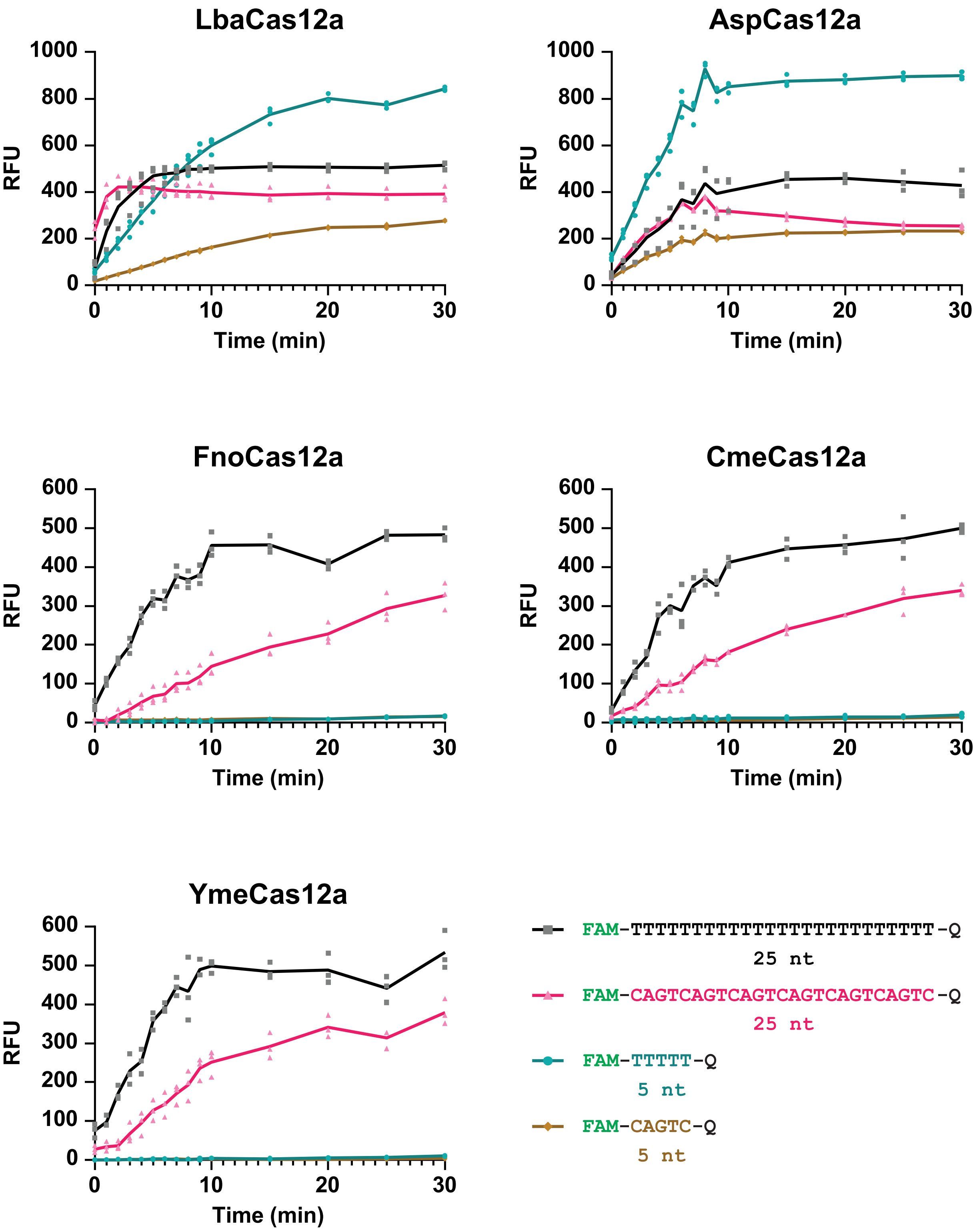

Single-stranded DNA reporters with a 5' fluorophore and 3' quencher were incubated with Target W crRNA-Cas12a RNPs and Target W DNA. Reactions with Lba, Asp and FnoCas12a were incubated

at 37 °C while reactions with Cme and YmeCas12a were incubated at 55 °C. Fluorescence was recorded over time and the mean relative fluorescent unit (RFU) values for reactions with 25 nt poly T (black), 25 nt CAGT (magenta), 5 nt poly T (blue), 5 nt CAGT (brown) reporters are plotted as lines. The RFU values represent background normalized signal derived by subtracting the fluorescence of negative control reactions that did not contain target DNA. Individual data points for all experimental replicates are plotted on the graphs and the source data can be found in the supplementary materials.

Figure S11. Cas12a *trans* nuclease activity acts on RNA in addition to DNA.

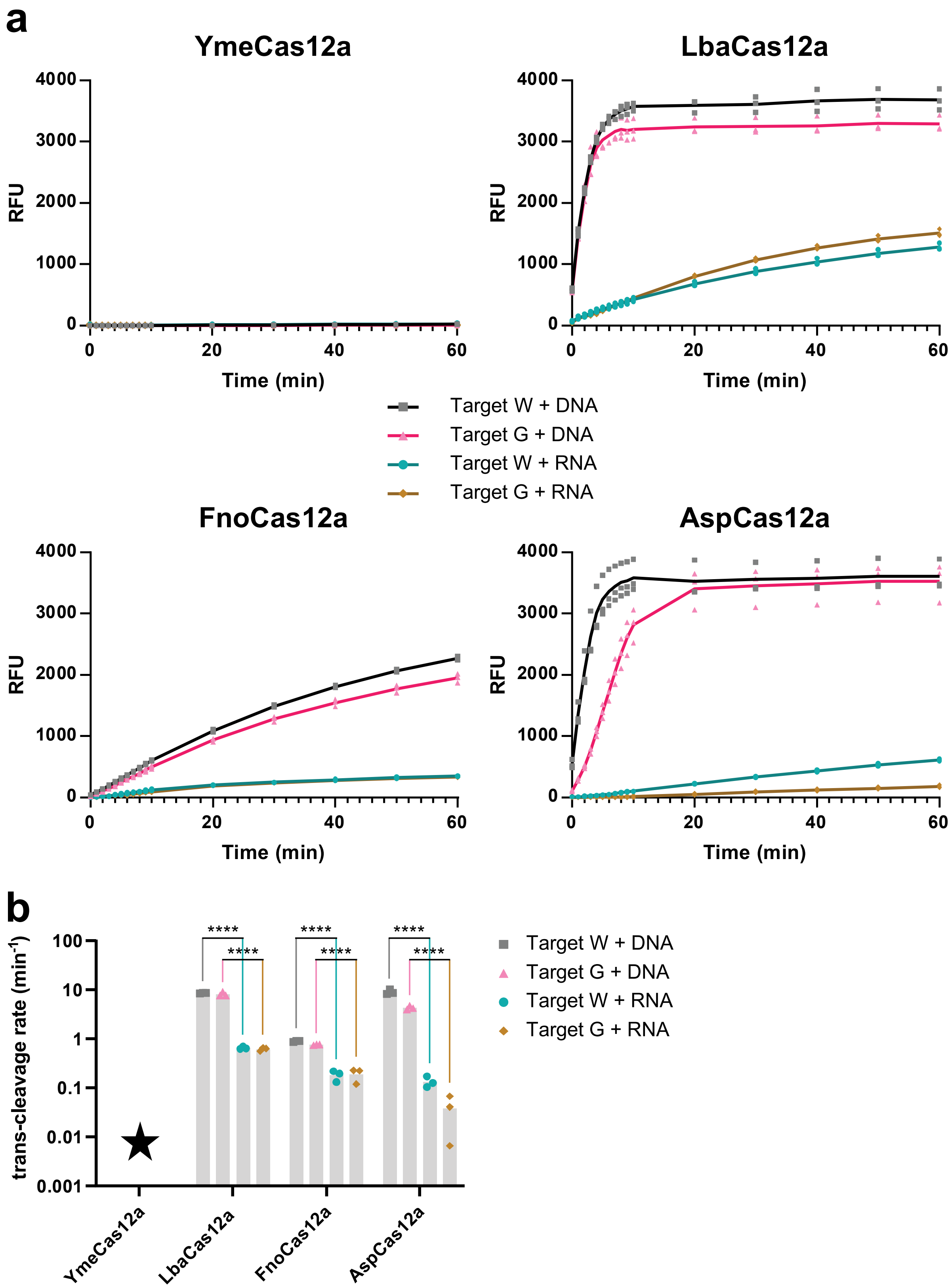

Trans nuclease activity of Cas12a RNPs was activated by incubation with double-stranded target DNA. A 5 nt randomized sequence reporter oligonucleotide with a 5’ fluorophore and 3’ quencher was added. **(a)** Fluorescence was measured over time at 37 °C for a reporter that consisted of either DNA or RNA bases. Mean relative fluorescent unit (RFU) values for reactions with Target W and DNA reporter (black), Target G and DNA reporter (magenta), Target W and RNA reporter (blue) and Target G and RNA reporter (brown) are plotted as lines. The RFU values represent background normalized signal derived by subtracting the fluorescence of negative control reactions that did not contain target DNA. A graph of a representative negative control reaction as well as the reporter oligonucleotides digested with micrococcal nuclease can be found in Supplementary Figure S12. Individual data points for all experimental replicates are plotted on the graphs and the source data can be found in the supplementary materials. **(b)** The initial rate of reporter oligonucleotide cleavage was calculated using linear regression and the first 5 min of data. Two-way ANOVA with Tukey’s multiple comparisons test was used to determine if the rates for DNA reporters were significantly different than RNA reporters (**** indicates p-value <0.0001). A rate could not reliably be determined for YmeCas12a using these reaction conditions (black star).

Figure S12. *Trans* nuclease activity of LbaCas12a requires a functional RuvC domain and is independent of crRNA processing activity.

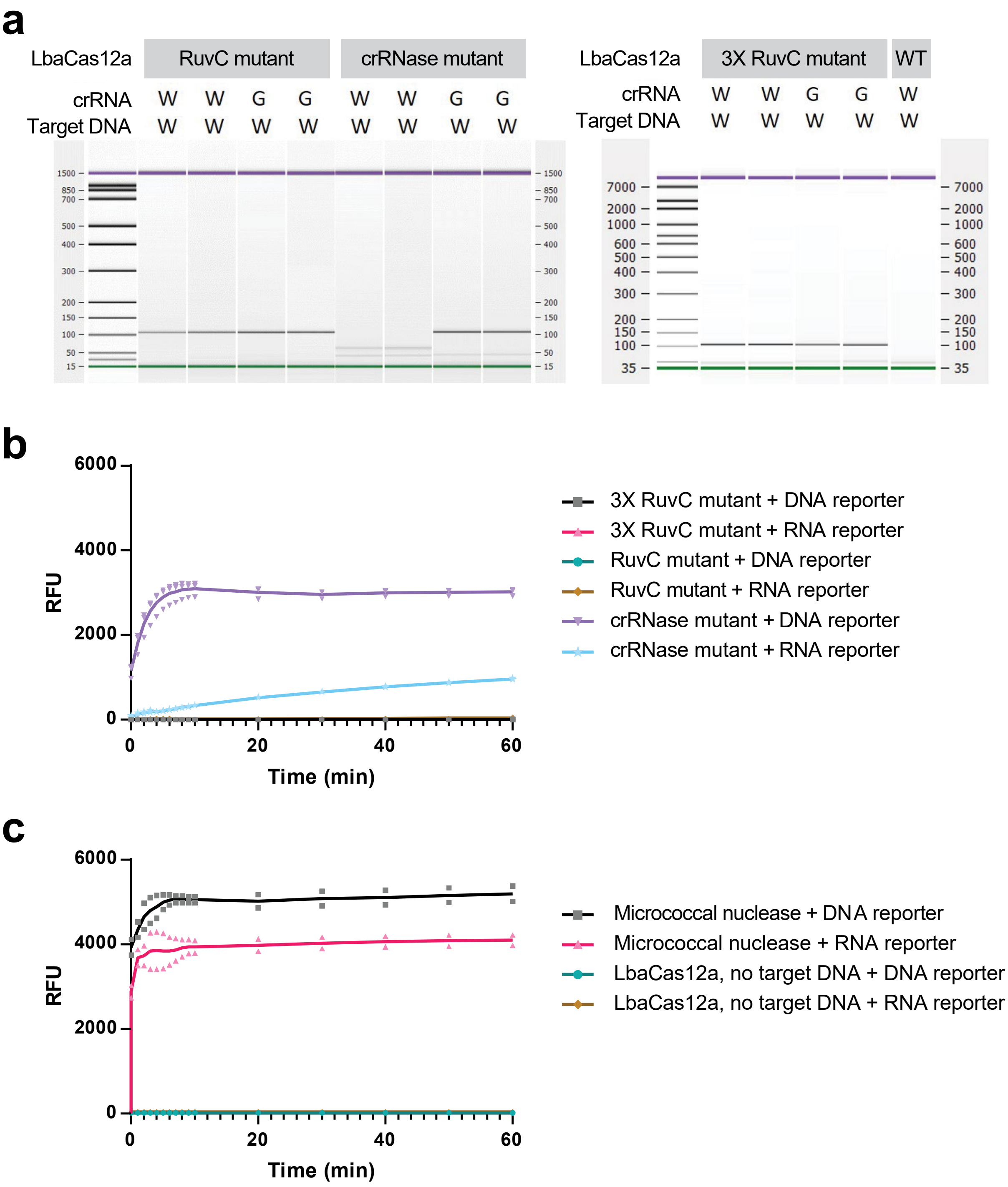

(a) Linear double-stranded Target W DNA was incubated with a crRNA-Cas12a RNP at 37 °C. The Cas12a RNPs consisted of a crRNA with a complementary (Target W) or non-complementary (Target G) sequence to the target DNA and either wild-type LbaCas12a (WT), LbaCas12a with a D832A

substitution in one of the three RuvC subdomains (RuvC mutant), LbaCas12a with D832A, E925A and D1180A substitutions in the RuvC subdomains (3X RuvC mutant), or LbaCas12a with an H759A substitution that inactivates the crRNA-processing RNase domain (crRNase mutation). Half of the sample was purified using a Monarch® PCR & DNA cleanup kit and analysed on an Agilent 2100 Bioanalyzer to assess cleavage of the target DNA. **(b)** To the remaining half of samples, a 5 nt randomized sequence reporter oligonucleotide consisting of DNA or RNA bases with a 5' fluorophore and 3' quencher was added. Fluorescence was measured over time and mean relative fluorescent unit (RFU) values for reactions are plotted as lines. The RFU values represent background normalized signal derived by subtracting the fluorescence of negative control reactions that did not contain target DNA **(c).** Individual data points for all experimental replicates are plotted on the graphs and the source data can be found in the supplementary materials.

Amino acid sequences used for protein expression and purification of Yme and CmeCas12a

YmeCas12a

```
MGMKIEEPKKKRKVSKVNNGFSSLCVWSDFTRKFSLSKTLRFELKPVGRTEDFLKQNKVFEKDKTIDD
SYNQAKFYFDKLHQKFINESLSPSSDSSNLKNIDLEYFAKQFLKLKSEIQKLKGEKKQKEANNKEKEI
NNLRKAYYKEIKSLLDKKAEEWKEIYKKKEIQFNETDLKQKGTDFLMKSGILGILKYEFPKEKEEELK
SKDWPSLFVEDKANPGDKVYIFDSFDDFATYLIKFQETRKNLYKDDGTSTAVATRVISNFEKFLNNKK
IFKDKYINYWKQIGITEGEKQIFEIDYYYNCFIQSGIDNYNDLIGKINQKSKQYRDKNKIEKSKLPLF
KVLDKQILGEVIKERELIIKTETETEEEVFINRFKEFIDQNKQRILRAQNLMNDLINEEFENEYSGIY
LKNSAINTIANRWFKNTTEFLLKLPQASKSKENKESPKVEPFVSLLDIKNALDDFEKEKDSLGTIFKD
KYYKTKESDEAPLNSDSQESYWKQFLKIWGYEFNQLFEDKFNEEGIKIFWGYTEDLEKQAENLNSFSR
KKEEIILVKNYCDATLRIYQMMKYFALEAKKQDDIPLAADCSPEFYNRFDEYYKDFKFIRYYDAIRNF
VTKKPSNEDKIKLNFESGSLLTGFDKNKESEKLGIILKNKNNNKYFLGIINKKHNKIFESKNENEFIG
NPAKDLYEKMELKLFPDPKKMIPKIAFADKNKKDFGWTQEIQKIKEDFGNFQENKKDSKDLKFDKNKL
SKLIEYYQNCLEKGNYKKEFDFEWKKPEEYQSMSEFNQDIEKKNYKIKFIPIKASYIDDKVKNGELYL
FEISNKDFIWPNQKKNIHTLYFLNLFSDKNIQKPVFRLGANAEVFYRPASVRKEMDKERSKGGKEIIK
YKRYTEDKMFLHLPIEINYGCPKAPNKNQYNKKIIEFLNKNKDEINIIGIDRGEKNLLYYTVINQKGE
ILDHGSLNEINGVNYFEKLIEREKERQINRQSWEPVVKIKDLKKGYLSYIVRKIADLVEKYNAIIVLE
DLNMRFKQVRGGIERSIYQQFEKQLIDKLGYLVFKDDRGPESPGGVLNGYQLLAPFTTFKDLGKQTGI
IFYTNAEYTSKTDPITGYRKNIYISNSASQKKIKENLINKLKEIGWDEKENSYFFTYNQKDFGSPISK
EWTLYSKVPRVIKENNNSTGYWEYKPIDLNEEFEDLFKKYGINEKSSDILSEIKELIKNNEGKLTRKQ
EFDGKNKNFYERFVYLFNLLLETRNTMSLRVKLDKRGNEIKLDEIDYGVDFFASPVKPFFTTAGVRFV
GRQIEGGKIQKEKKEEFTIKNFSDFERLFKNCPSDGFDSDGVGAYNIARKGIMILERIKQNPKNPDLY
ISKDDWDSFVLRNLSPKKKRKVLEHHHHHH
```

CmeCas12a

MGMKIEEPKKKRKVYKSTQQFTKLYSLSKTLRFELEPIGKTSYFIEKKGIISEDKLRSENYQKMKNTI
DGFHKYFIELAMENVHLTQLETFQKLYYASPEEKKNENFKKQFEEIQKKLRKEIADGFKTGKAKEIFS
KINKKELITELLEDWVQKQPNKQYYFDPSFKTFTTYFTGFNENRQNMYTDKVQSTAIAYRMIHENLPK
FLDNIKIFEQIKGIPELYEKSKTLFKNIEEFLNIKTIDEAFELPYFNEILTQKQIDIYNLIIVGRIAE
EGKPKIQGLNEYINLYNQLQKEKNKRVPKLKVLYKQILSDREKTSFTIDKFENSQEVTEAINGFYHHN
IISYKPADKPEPENILETLEELLSSLKEYSLDKIYLRNDNQLTQISQKIFGNFSVFLDALSYYYDKVI
EPNFENDYQKANEKKKEKLDAEKNKYLKQDYFSIAHLQYALDNYVLVIDDSVEWKEKYSTSCIADYFK
THYKANKKEDSSKEFSFTADIQSKYSCIKGLLENYPMDKKLHQDKQSIDNIKLFLDSIIELLHFVKPL
ILATDSTLEKDQNFYGQLEPWFESLKEIIRLYNKVRNFATQKAYSTEKFKLNFDNSTLLDGWDINKET
DNLGILFIKGHDYYLGIMDKKHNRIFKSIPAPNTDRTYQKVNYKLLPGPSKMLPKVFFSKSNIEYYRP
SEEILRIRNHSTHTKNGKPQDGFEKKDFNLEDCHAMIEFFKQSIEKHPEWNNFGFKFSNTKSYKSIDE
FYQEVENQGYYLNFTEVDEDYVHQLIDDGKLYLFRIKNKDFSTHSKGKPNLHTLYWKALFSPENLKDV
VYKLNGQAEIFYRKSSIKPENKIIHKANQTIGNKNPLAKKKQSIFTYDIIKDKRFTVDKYQFHVPITL
NFKSKGSDNINYEVLNYLKQNNKDVNIIGLDRGERHLIYLTLINQKGQIIHQESLNTIKSDNFEIETP
YHELLIQKENERDEARKSWGTIENIKELKEGYLSQVVHKIAKMMVEHNAIVVMEDLNMGFKRGRFKVE
RQVYQKLEKMLIDKLNYLIFKDFHQNEPGGLFHALQLTSKFESFKKMGKQSGFLFYVPASYTSKIDPE
TGFVNLFQTKYENIEKAKSFFQKFKSIRYNKEQHYFEFEFDYNDFTTKAEGTKNKWTICTYGERILTF
RNTAKNNQWDNQTVMLTEQFEDLFGKYNIVYGDGKDLKEKICEQNDKDFFKTMLNLFKLTLQMRNSIT
GTDEDFLISPVRNTRGEFFDSRKTDHFLPQNADANGAYHIAKKGLWWIKQIKEFNDNDWKKLNLDKTN
KGWLKFVQEPKKKRKVLEHHHHHH

Key:

SV40 Nuclear localization sequence

His-tag

Cas12a sequence
