# Supplementary material for: Characterization of Cme and Yme thermostable Cas12a orthologs
Source: Commun Biol. 2022 Apr 6;5:325. doi: 10.1038/s42003-022-03275-2 (PMC8986864; doi:10.1038/s42003-022-03275-2)
Supplement: Supplementary file 3 — Description of Additional Supplementary Files [file 42003_2022_3275_MOESM3_ESM.pdf]

Description of Additional Supplementary Files

File name: Supplementary Data 1
Description: Oligonucleotide sequences used in the study.

File name: Supplementary Data 2
Description: Source data used to make graphs and figures.
