# Supplementary material for: Characterization of Cme and Yme thermostable Cas12a orthologs
Source: Commun Biol. 2022 Apr 6;5:325. doi: 10.1038/s42003-022-03275-2 (PMC8986864; doi:10.1038/s42003-022-03275-2)
Supplement: Supplementary file 6 — Reporting Summary [file 42003_2022_3275_MOESM6_ESM.pdf]

nature research

Corresponding author(s): Ryan T. Fuchs, Jennifer L. Curcuru, Megumu Mabuchi, Audrey Noireterre, Peter R. Weigele, Zhiyi Sun, and G. Brett Robb

Last updated by author(s): Feb 17, 2022

Reporting Summary

Nature Research wishes to improve the reproducibility of the work that we publish. This form provides structure for consistency and transparency in reporting. For further information on Nature Research policies, see our Editorial Policies and the Editorial Policy Checklist.

Statistics

For all statistical analyses, confirm that the following items are present in the figure legend, table legend, main text, or Methods section.

n/a	Confirmed	
☐	☒	The exact sample size (*n*) for each experimental group/condition, given as a discrete number and unit of measurement
☐	☒	A statement on whether measurements were taken from distinct samples or whether the same sample was measured repeatedly
☐	☒	The statistical test(s) used AND whether they are one- or two-sided<br>*Only common tests should be described solely by name; describe more complex techniques in the Methods section.*
☒	☐	A description of all covariates tested
☒	☐	A description of any assumptions or corrections, such as tests of normality and adjustment for multiple comparisons
☒	☐	A full description of the statistical parameters including central tendency (e.g. means) or other basic estimates (e.g. regression coefficient) AND variation (e.g. standard deviation) or associated estimates of uncertainty (e.g. confidence intervals)
☒	☐	For null hypothesis testing, the test statistic (e.g. *F*, *t*, *r*) with confidence intervals, effect sizes, degrees of freedom and *P* value noted<br>*Give P values as exact values whenever suitable.*
☒	☐	For Bayesian analysis, information on the choice of priors and Markov chain Monte Carlo settings
☒	☐	For hierarchical and complex designs, identification of the appropriate level for tests and full reporting of outcomes
☒	☐	Estimates of effect sizes (e.g. Cohen's *d*, Pearson's *r*), indicating how they were calculated

Our web collection on statistics for biologists contains articles on many of the points above.

Software and code

Policy information about availability of computer code

Data collection	*Provide a description of all commercial, open source and custom code used to collect the data in this study, specifying the version used OR state that no software was used.*
Data analysis	Custom code was used to analyze the Illumina sequencing results. The code has been published and is referenced in our document. Reference #72. Sun, Z. and Robb, G.B. 2020

For manuscripts utilizing custom algorithms or software that are central to the research but not yet described in published literature, software must be made available to editors and reviewers. We strongly encourage code deposition in a community repository (e.g. GitHub). See the Nature Research guidelines for submitting code & software for further information.

Data

Policy information about availability of data

All manuscripts must include a data availability statement. This statement should provide the following information, where applicable:

- Accession codes, unique identifiers, or web links for publicly available datasets
- A list of figures that have associated raw data
- A description of any restrictions on data availability

Data from experiments involving high-throughput sequencing have been deposited in the NCBI Sequence Read Archive under accession number PRJNA718241. Supplementary Data are available online: Supplementary Table S1 and Supplementary Figures S1-S12 and Source Data

April 2020

Field-specific reporting

Please select the one below that is the best fit for your research. If you are not sure, read the appropriate sections before making your selection.

☒ Life sciences ☐ Behavioural & social sciences ☐ Ecological, evolutionary & environmental sciences

For a reference copy of the document with all sections, see nature.com/documents/nr-reporting-summary-flat.pdf

Life sciences study design

All studies must disclose on these points even when the disclosure is negative.

Sample size	Quantitative analyses were replicated at least 2 times, most were replicated 3 times and up to 6 times independently. Experiments involving Illumina sequencing were typically performed once, however a similar experiment was performed with an alternate target sequence. These experiments produced similar results and so act as a quasi-replicate.
Data exclusions	No data was excluded from the analysis.
Replication	All attempts at replication were successful. Some experiments were repeated more than 3 times to ensure confidence in the results. We will provide raw data that has all data points that were used to make the heat maps and graphs in our figures.
Randomization	This is not applicable because we don't have experimental groups in this study.
Blinding	Blinding was not incorporated into our study. All samples were processed identically and all attempts were made to be accurate and precise.

Reporting for specific materials, systems and methods

We require information from authors about some types of materials, experimental systems and methods used in many studies. Here, indicate whether each material, system or method listed is relevant to your study. If you are not sure if a list item applies to your research, read the appropriate section before selecting a response.

Materials & experimental systems

n/a	Involved in the study	
☒	☐	Antibodies
☒	☐	Eukaryotic cell lines
☒	☐	Palaeontology and archaeology
☒	☐	Animals and other organisms
☒	☐	Human research participants
☒	☐	Clinical data
☒	☐	Dual use research of concern

Methods

n/a	Involved in the study	
☒	☐	ChIP-seq
☒	☐	Flow cytometry
☒	☐	MRI-based neuroimaging
